# Perception of Gingival Bleeding by People and Healthcare Professionals: A Multicentre Study in an Adult French Population

**DOI:** 10.3390/ijerph17165982

**Published:** 2020-08-18

**Authors:** Alexandre Baudet, Thomas Veynachter, Hélène Rousseau, Fani Anagnostou, Sylvie Jeanne, Valérie Orti, Nathalie Thilly, Céline Clément, Catherine Bisson

**Affiliations:** 1 Faculty of Dentistry, University of Lorraine, 54505 Vandœuvre-lès-Nancy, France; thomas.veynachter@univ-lorraine.fr (T.V.); celine.clement@univ-lorraine.fr (C.C.); catherine.bisson@univ-lorraine.fr (C.B.); 2 Department of Dentistry, University Hospital, 54000 Nancy, France; 3 Platform Support for Clinical Research, University Hospital of Nancy, 54500 Vandoeuvre-Lès-Nancy, France; h.rousseau@chru-nancy.fr (H.R.); n.thilly@chru-nancy.fr (N.T.); 4 Department of Periodontology, University Hospital and University of Diderot, 75006 Paris, France; fani.anagnostou@univ-paris-diderot.fr; 5 Department of Periodontology, University Hospital and University of Rennes 1, 35000 Rennes, France; sylvie.jeanne@univ-rennes1.fr; 6 Department of Periodontology, University Hospital and University of Montpellier, 34193 Montpellier, France; valerie.orti@umontpellier.fr; 7 Laboratory “Interpsy”, University of Lorraine, EA 4432, CEDEX 54015 Nancy, France; 8 Laboratory “Health Systemic Process”, University Lyon 1, EA 4129, 69008 Lyon, France

**Keywords:** gingival bleeding, gingivitis, periodontal diseases awareness, self-report, reaction, oral hygiene, healthcare professional

## Abstract

Gingival bleeding (GB) is a common sign of gingival inflammation, which indicates the presence of periodontal diseases. This study aimed to describe the perception of French adults about their self-reported GB and answers of healthcare professionals regarding the GB reported by these interviewees. A questionnaire administered by one investigator in each of three public settings of four cities in France from September 2016 to November 2017. Among 794 adults interviewed, 502 (63.2%) reported a GB. Among them, 414 (82.5%) believed that GB is benign, and 309 (61.6%) declared one or more responses. The three main responses were to use mouthwash (29.3%), to change to a soft-bristle toothbrush (20.1%) and to modify the brushing technique (19.3%). Almost half (49.0%) questioned at least one healthcare professional concerning their GB: a dentist (43.0%), a physician (14.1%), and a pharmacist (8.0%). The main response of each healthcare professional was: for dentists: a “prescription of mouthwash”, for physicians to say “gingival bleeding is not serious”; and for pharmacists: “to sell a mouthwash”. Most of the participants considered their GB as benign and had inappropriate responses, which indicates their lack of knowledge regarding periodontal health. The same conclusions can be drawn for healthcare professionals, as reported by interviewees.

## 1. Introduction

Gingival bleeding (GB) is the most easily identifiable sign of gingivitis. It may also indicate the presence of periodontitis. Periodontal diseases are one of the most common human pathologies. According to the global burden of Oral Conditions 2010 study, the disability-adjusted life-years due to severe periodontitis increased between 1990 and 2010 [1]. This pathology is a public health problem because it is widespread and impairs the oral and general well-being of people [2].

GB is a reversible inflammation of the gingiva caused by the accumulation of dental plaque, which may include more than 700 different species of bacteria [3,4]. Among these bacteria organized inside a biofilm, certain micro-organisms present numerous virulence factors leading to the degradation of the tissue supporting the teeth and may lead to tooth loss. These pathogenic bacteria and their metabolic by-products have not only a local effect but may also modulate the immune response beyond the oral cavity, and thus promote the development of systemic conditions after entering in bloodstream [5,6]. Indeed, among pathogens in periodontal diseases “keystone pathogens” such as *Porphyromonas gingivalis*, *Tannerella forsythia*, *Treponema denticola,* and *Aggregatibacter actinomycetemcomitans* have been detected in blood, coronary atheromatous plaque, placenta, and brains of Alzheimer’s disease patients [7,8,9,10]. Therefore, periodontitis has been associated with various systemic diseases such as cardiovascular disease, diabetes, adverse pregnancy outcomes, Alzheimer’s disease, oral and colorectal cancers, respiratory tract infection, bacterial pneumonia, and rheumatoid arthritis [7,11,12,13,14,15]. The global cost of periodontal diseases is not negligible and the knowledge of their etiology by patients and healthcare professionals is very important in order to prevent them and to promote its early treatment.

In France, the last nationwide clinical study of periodontal status on a representative sample of the population was conducted among 2144 adults aged 35–64 years in 2002–2003. It showed only results concerning the attachment loss and the probing depth, but no information on the gingival status [2]. Among all the studies investigating periodontal diseases, very few of these reported the attitudes and reactions of patients regarding the GB. For example, Dong et al. [16] carried out a qualitative study about perceptions of GB among Chinese immigrants in Montreal. Moreover, the evaluation of awareness and knowledge of periodontal diseases among healthcare professionals is also very rare. To our knowledge, no publication studied the perception and the reactions of both patients and healthcare professionals to GB.

The main objective of this study was to describe the perception, attitude, and oral health practices among a representative sample of French dentate adults who self-reported GB. The secondary objective was to determine the perception and responses of healthcare professionals questioned about GB reported by patients declaring a GB.

## 2. Materials and Methods

### 2.1. Study Design

A multicenter cross-sectional study was conducted in four major urban centers in France: Montpellier, Nancy, Paris, and Rennes. Data were collected between September 2016 and November 2017 with a questionnaire administered by four investigators calibrated by their local chief academic.

### 2.2. Participants, Settings, and Administration of the Questionnaire

The population studied comprised a representative sample of dentate adults (≥18 years) living in four administrative regions of France:

- North region, with the city of Paris, the French capital (2,206,000 inhabitants, the first French city)
- South region, with the city of Montpellier (278,000 inhabitants, the seventh French city)
- West region, with the city of Rennes (215,000 inhabitants, the eleventh French city)
- East region, with the city of Nancy (105,000 inhabitants, the forty-second French city)

In each administrative region, three public settings were investigated: a center for preventive medicine (100 people by center), a railway station (50 people by station), and a mall (50 people by center). These sites were chosen in order to interview adult people who are representative of the French population regarding the age, sex, socioeconomic status, and education level.

The questionnaires were administered for a few days from 9 a.m. until 7 p.m. by four dental students: one investigator per region. Each interview lasted approximately 10 min per subject.

To be included, the subjects had to be at least 18 years old, have at least 20 remaining teeth, and accepting to respond to the questions after explanation of the survey’s purpose. The recruitment was based on a convenience sample. Participation was voluntary, anonymous, and without any compensation. Anonymity was guaranteed at all phases of data collection and analyses. According to the French legislation, no ethical approval was necessary when the survey questionnaire was developed.

### 2.3. Survey Instrument

The questionnaire consisted of close-ended questions divided into five sections. The first collected sociodemographic data (age, sex, level of education, profession). The second investigated oral health behavioral data (brushing technique, frequency, and bristle hardness of toothbrush). The third explored the GB: presence (yes/no) and trigger factor (spontaneously/during teeth brushing). The fourth assessed dental trait anxiety perceived by the patient interviewed using the dental anxiety scale (DAS) [17,18], which has been shown to be one of the more inclusive, highly validated, and reliable scales. Responses to the four items of the scale were scored from 1 to 5, producing total scores ranging from 4 to 20. Individuals were considered as not anxious for a DAS score <9, moderately anxious for a DAS score between 9 and 12, and highly/severely anxious for a DAS score ≥13 [17]. The last section of our questionnaire investigated the awareness, the perception and the reactions of the respondents to their GB as well as the reactions of their healthcare professionals reported by the patients regarding the GB. The data from this last section and its relation with sociodemographic factors were the only data analyzed in this article.

### 2.4. Statistical Analyses

The sample size was calculated with respect to the multiple logistic regression model which was used in the statistical analyses. According to Peduzzi et al. [19], the minimum number of cases to include is equal to N = 10 k/p, k being the number of covariates in the regression model and *p* being the smallest of the proportions of positive or negative cases in the population (here the prevalence of GB). With k = 20 (the number of covariates in the regression model) and *p* = 0.30 (the most common prevalence of GB found in the literature), *n* = 666. Considering the proportion of potentially erroneous or incomplete questionnaires, the estimated sample size was 800. Therefore, we included 200 subjects in each of the four towns.

All data were collected on Microsoft^®^ Excel (Microsoft Corporation, Redmond, WA, USA) and analyzed using SAS^®^ software version 9.4 (SAS Institute Inc., Cary, NC, USA). Data were described as numbers and percentages for categorical variables and as mean ± standard deviation (SD) for continuous variables. Missing data were not imputed. Six questionnaires were excluded because they were insufficiently completed. Univariate analysis of categorical variables were performed with a chi-square test or Fisher’s exact test when expected frequencies were <5. The significance level was fixed to *p* < 0.05.

## 3. Results

### 3.1. Characteristics of Participants

In this study, 794 adults answered the questionnaire properly: 399 (50.3%) in preventive medicine centers, 198 (24.9%) in shopping centers, and 197 (24.8%) in railway stations. Among the interviewees, 418 were females (52.6%) and 376 were males (47.4%), with 44.0 ± 17.2 years-old (18 to 86 years) (Table 1). There was no significant difference between the sample vs. the French population for age distribution [20]: 46.2% vs. 43.4% for the 18–40 years, 33.6% vs. 34.7% for the 41–60 years and 20.2% vs. 21.9% for the >60 years age group. There were 52.6% of females in the study group and 52.5% in the French adult population [20]. There was also no significant difference between the sample vs. the French population for the occupational categories [21]: retailers (5.4% vs. 6.0%), executives (17.0% vs. 16.1%), administrative (23.4% vs. 24.6%), employees (29.2% vs. 28.5%), manual workers (18.8% vs. 21.9%), others (5.5% vs. 1.4%).

Among 794 respondents, 502 (63.2%) reported GB, which included 466 (58.7%) only during teeth brushing and 36 (4.6%) spontaneously (data not shown).

### 3.2. Perception and Responses to the GB among Population GB Positive

**Figure 1:**
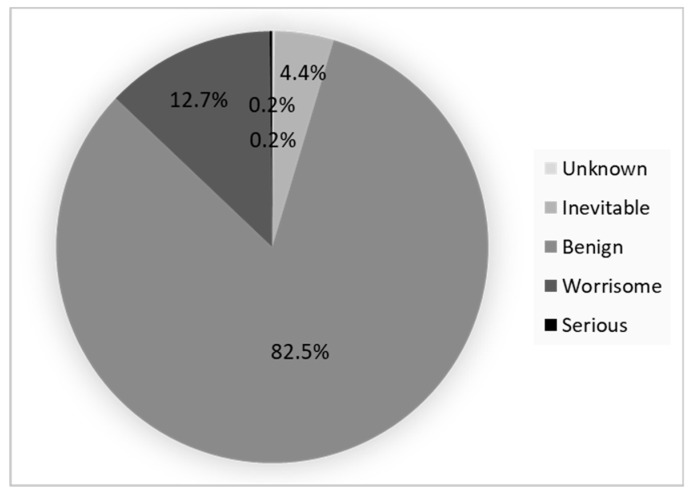
Perception of GB among respondents with self-reported gingival bleeding.

Among the 502 responders GB positive, 414 (82.5%) declared that their GB was benign, 64 (12.7%) worrisome, and 22 (4.4%) inevitable. Only one respondent (0.2%) believed that this could be a serious symptom, and one (0.2%) had no opinion (Figure 1).

The types of responses according to sociodemographic characteristics of respondents reporting GB are described in Table 2. Among responders that were GB positive, 309 (61.6%) had one or more responses distributed as follows concerning the management of their GB: 147 (29.3%) used mouthwash, 101 (20.1%) changed for a soft-bristle toothbrush, 97 (19.3%) modified their brush technique, 80 (15.9%) made an appointment with a dentist and 21 (4.2%) stopped tooth-brushing. Moreover, females declared significantly more responses to their bleeding than males: 189/278 (68.0%) female vs. 120/224 (53.6%) male (*p* = 0.001). Anxious respondents declared significantly more responses: 78/119 (65.5%) respondents with severe anxiety, 89/130 (68.5%) with moderate anxiety and 142/253 (56.1%) without anxiety reacted to their bleeding (*p* = 0.037). The response “stop brushing” was significantly associated with the male group (*p* = 0.007) and anxiety-level classes (*p* = 0.002). The different age groups of respondents were significantly associated with the use of mouthwash (*p* = 0.03) and with the reaction “appointment with a dentist” (*p* = 0.04).

### 3.3. Healthcare Professionals Responses to the GB Reported by Their Patient

**Figure 2:**
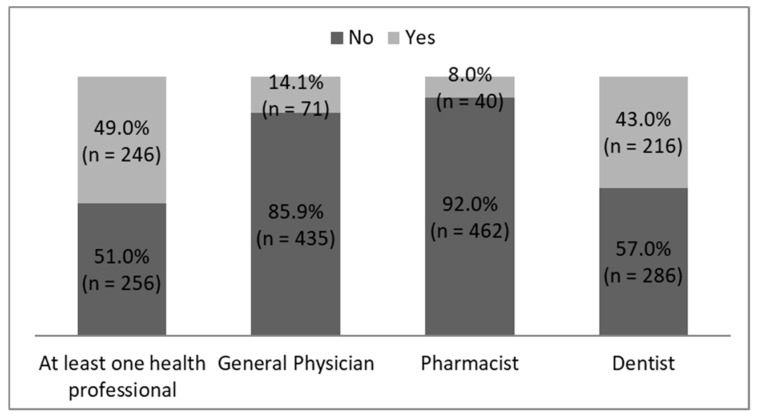
Percentage and number of healthcare professionals questioned about bleeding by respondents with gingival bleeding.

The percentage and number of healthcare professionals questioned about bleeding by respondents GB positive are described in Figure 2.

The interviewees reported different types of reaction from healthcare professionals which have been questioned about their GB during a consultation (Table 3). According to GB positive patients, among the dentists questioned, 40.7% have examined the gingiva of the patient, 55.1% have prescribed appropriate mouthwash or toothpaste, but only 38.4% have trained their patients in tooth brushing technique. Moreover, 2.8% of dentists and 5.6% of physicians have explained that GB is a common and normal phenomenon. According to patients GB positive, among the general physicians questioned, 28.2% have prescribed appropriate mouthwash or toothpaste and 21.1% a toothbrush with soft bristles, 15.5% educated the patient regarding tooth brushing technique and finally, 26.8% referred the patient to a dentist. According to GB positive patients, among the pharmacists questioned, 45.0% have sold mouthwash or toothpaste and 30.0% a toothbrush with soft bristles to the patient reported GB and 25.0% advise them to make an appointment to the dentist. According to all the respondents, 47.9% of their physicians, 32.5% of their pharmacists, and 27.3% of their dentists believed that “bleeding is not serious”. 

No statistical difference was observed between the respondents of the different centers regarding their reaction to their GB. Only interviewees from the railway station and mall have more often questioned their general physician regarding this symptom (*p* < 0.04).

## 4. Discussion

Even if the clinical examination of periodontal parameters remains the gold standard for the prevention and/or management of periodontal diseases, the self-report could be an interesting inexpensive, less time and less resource-consuming alternative which could facilitate epidemiologic studies on a much larger scale, and reflect the patient’s perception of their own oral health status [22]. To our knowledge, the present study is the first study that evaluated the perception of people to their own self-reported GB and the attitudes of healthcare professionals regarding GB reported by the people declaring GB. Indeed, information/data on oral health, like gingivitis prevalence, is essential for developing, implementing, and evaluating public health interventions for oral diseases. The diagnosis of gingivitis is crucial to prevent or limit the development of a more destructive periodontal disease such as periodontitis [1,23].

GB is one of the major signs of gingivitis. However, this symptom is considered as benign by 82.5% of the French population interviewed in our study through ignorance. The patients’ lack of awareness of GB associated problems is a public health concern because it is one of the first clinical signs of periodontal disease. As shown in the Gift review, many individuals in the USA do not recognize the symptoms of periodontal disease and do not associate these symptoms with a disease [24]. Conversely, a questionnaire evaluating the periodontal knowledge among adults in Jordan showed that the majority of participants were aware that GB indicated the presence of periodontal diseases [25]. In our study, 61.6% of adults reporting GB have responded to this bleeding, which seems normal because gingivitis is generally associated with pain, difficulties in performing oral hygiene [26], and impacts negatively the oral health-related quality of life [27]. In our study, the fact that people reported GB and looked for a solution to reduce or stop it showed that they had taken it into account. The most frequent responses were to rinse with a mouthwash, followed by the purchase of soft-bristle toothbrush, the modification of the brushing technique, and booking a dental appointment. Mouthwash with an antiseptic agent such as chlorhexidine can reduce gingivitis and plaque, but it is only an adjunctive treatment for gingival health [28,29]: the mechanical oral hygiene is essential [30]. Indeed, as shown in cellular models, the exopolymeric matrix inhibits or limits the penetration of antiseptic molecules inside the biofilm, preventing bacterial destruction [31]. The change of toothbrush for a soft-bristle toothbrush is interesting but not sufficient [32]. The effectiveness of the tooth brushing depends on the type of toothbrush [33], brushing technique [34], time and frequency of brushing [35], and type of interdental cleaning devices [36]. Using calibrated interdental brushes daily is necessary to reduce the periodontal pathogens [37] and the interproximal bleeding [38], especially since interdental inflammation and periodontal pathogens in the interdental space can go unnoticed in healthy subjects, without clinical signs of gingivitis [39].

Finally, the best adequate responses were to change the brushing technique, which was obviously inefficient, to use interdental brushes and to make a dental appointment. Nevertheless, among GB positive respondents, only 15.9% consulted a dentist for this problem, which is far from satisfactory. Indeed, the dentist is the only one to be able to make a periodontal diagnosis and to offer professional preventive measures and care [40]. Individualized oral hygiene practices that optimize plaque control and reduce the gingival inflammation should be employed and reinforced by dental professionals [41]. Thanks to an appropriate method, the patients would easily remove all supra-gingival biofilm and control the gingival inflammation. Hygienists also understand the primary prevention of periodontal diseases, but this profession does not exist in France. Moreover, some responses and attitudes of people reporting GB were not at all appropriate: 38.4% of our respondents declared no reaction against their GB and 4.2% stopped tooth-brushing. This last response is significatively more self-reported by very anxious people and by males (*p* < 0.01). The absence of reaction can be explained by the fact that some people perceived the GB as a common and almost normal phenomenon [42]. Globally, all these responses showed the lack of knowledge regarding periodontal diseases and the need to inform the population about the cause of the GB and its potential impact on systemic diseases. Indeed, the principal cause of the GB is the presence of bacteria on the teeth [43]. The bacteraemia induced by a spontaneous bleeding or after toothbrushing could have an impact on systemic diseases such as diabetes, cardiovascular disease, rheumatoid arthritis, and Alzheimer’s disease [44]. Consequently, these physicians (cardiologists and endocrinologists) are already aware of the impact of some oral bacteria in diseases regarding their expertise area, which is obviously not the case for general practitioners and pharmacists interviewed in this study.

GB can be the symptom of both gingivitis and periodontitis which are infectious diseases, so the risk of bacteria, potentially keystone pathogens of periodontal diseases, entering the bloodstream must not be underestimated. Regarding healthcare professional responses to the GB, the interviewees reported that their general physician declared: “this bleeding is not serious” and “bleeding is common and normal phenomenon”. According to the interviewees, dentists and pharmacists replied in a lower frequency these same responses, which probably reflects a poor knowledge of the relationship between periodontal diseases with systemic diseases. The prescription of mouthwash is “natural” for pharmacists, but it is less normal for a dentist, because the only way to health with GB is to remove dental plaque; the mouthwash is only an adjuvant. According to the responses reported by the participants, French general dentists seem to show a low interest in the etiology, prevention, and treatment of periodontal diseases. The periodontal awareness of periodontal disease varied according to the country. In parallel with the increase of the standard of living, the patients’ expectation of the skills of all professionals, including dentists, has undoubtedly risen. The lack of any medical procedure registered in social insurance for dental hygiene could partially explain to a certain extent that not all the dentists have taken the time to educate their patients on an efficient tooth brushing technique, and, if necessary, to choose interdental cleaning devices.

Only a quarter of general physicians and of pharmacists had advised their patients to consult a dentist, which could finally be in accordance with the belief of the bleeding innocuity. These healthcare professionals were less implicated in oral hygiene. But interestingly, some physicians have taken time to educate patients to brush their teeth while no pharmacist proposed it. It seems important that all healthcare professionals must deliver appropriate messages to their patients regarding GB and its potential effect on certain systemic diseases.

The awareness of oral-systemic disease relationship has been investigated among French general physicians. The answers to the questionnaires showed that French physicians were informed about the association between periodontitis with cardiovascular diseases and diabetes. Other pathologies, such as respiratory infection, obesity, and joint diseases are more weakly known. In the conclusion of both studies, general physicians considered their insight of periodontal diseases as insufficient. But undoubtedly, there is a gap in the understanding of the mechanism of periodontal disease, in particular the bacterial etiology of the bleeding, even if they were aware of some relations oral-systemic diseases [45,46]. No study has evaluated the knowledge of oral health and oral-systemic disease relationship among French pharmacists. By contrast, two studies have investigated the attitude and practice toward oral healthcare of Saudi Arabian and Lebanese pharmacists [47,48]. According to Lebanese pharmacists, one of the most reported oral conditions was GB, and 85.5% of them have declared to provide good oral health advice to their patients regarding this symptom [47]. In a Saudi Arabian study, oral health advice regarding GB was also requested by the patient to pharmacists; the recommendations given were based on the personal experiences of the pharmacist and responses from other patients [48]. No information about the response of the pharmacists was detailed in both studies. Finally, a majority of pharmacists interviewed were keen on improving their knowledge regarding oral health during their academic study and/or by training programs. Indeed, pharmacists believed that they play an important role in the improvement of community oral health and even in the promotion of oral health preventive programs.

The limit of this study was the self-reporting of all the different responses of (i) people declaring GB and (ii) healthcare professionals questioned about bleeding by the patient’s self-reported bleeding. They could not be exactly the real responses of each professional, but nevertheless, these reflect people’s perception and understanding of the professionals’ responses. However, the global responses of all professionals showed the lack of knowledge regarding the local impact of GB, symptoms of periodontal diseases, and the potential effect of oral bacteremia on certain systemic diseases. This self-reported questionnaire could be an inexpensive and useful alternative to promote dental hygiene and to strengthen the participation of all healthcare professionals regarding this symptom and its systemic effect. The self-perception of this bleeding could be an interesting approach to support the behavior changes of the patient regarding their oral hygiene. A periodontal awareness program could set up in France aimed at improving periodontal health, through increasing the knowledge of both public and professionals. The European Federation of Periodontology organizes the “Gum health day” each year in order to raise public awareness of the importance of identifying GB in many countries. But this initiative takes place only for one day through videos shown over the internet and local actions in cities with dental universities, which is far from sufficient to promote oral health and encourage people to visit their dentist for a periodontal check-up. A larger periodontal campaign and the enhancement of the knowledge of periodontal diseases through educational and continuous training programs for healthcare professionals are needed to really promote the prevention of these diseases.

## 5. Conclusions

The self-reported GB is perceived as benign by a majority of French people and as “not serious” by many healthcare professionals. Many responses of these people were inappropriate or not optimal regarding the control of the GB. The prevention of periodontal diseases needs a better awareness of both public and healthcare professionals thanks to national campaigns and appropriate educational programs, respectively.

## Figures and Tables

**Table 1:** Sociodemographic characteristics of all the respondents and respondents with self-reported gingival bleeding.

Characteristics of Respondents	All Respondents		Self-Reported Gingival Bleeding	
	*n*	%	*n*	%
All	794	100.0	502	63.2
Center				
Preventive medicine	399	50.3	283	70.9
Mall	198	24.9	99	50.0
Railway station	197	24.8	120	60.9
Sex				
Female	418	52.6	278	66.5
Male	376	47.4	224	59.6
Age group (years)				
18–40	367	46.2	260	70.8
41–60	267	33.6	169	63.3
>60	160	20.2	73	45.6
Level of education				
Low	280	35.3	179	63.9
Medium	143	18.0	93	65.0
High	371	46.7	230	62.0
Profession/occupations				
Unemployed/student/ other	233	29.3	165	70.8
worker/salaried/artisan/merchant/official	301	37.9	205	68.1
executive/teacher/ liberal profession	119	15.0	65	54.6
Pensioner/retired	141	17.8	67	47.5
Level of dental anxiety				
None	438	55.2	253	57.8
Moderate	197	24.8	130	66.0
High	159	20.0	119	74.8

Note: low level: primary education (8 years or less), medium level: some secondary education (9–11 years), high level: completed secondary education (12 years or more).

**Table 2:** Types of responses according to sociodemographic characteristics of respondents reporting gingival bleeding.

Characteristics of Respondents	Mouthwash			Soft-Bristle Toothbrush			Brushing Technique Modification			Dental Appointment			Stop Brushing		
	*n*	%	*p*	*n*	%	*p*	*n*	%	*p*	*n*	%	*p*	*n*	%	*p*
All	147	29.3		101	20.1		97	19.3		80	15.9		21	4.2	
Sex			1.0			0.1			0.2			0.3			0.007
Male (*n* = 120)	57	47.5		33	27.5		33	27.5		35	29.2		14	11.7	
Female (*n* = 189)	90	47.6		68	36		64	33.9		45	23.8		7	3.7	
Age group (years)			0.03			0.6			0.6			0.04			0.1
18–40 (*n* = 158)	66	41.8		51	32.3		53	33.5		33	20.9		14	8.9	
41–60 (*n* = 107)	53	49.5		33	30.8		33	30.8		37	34.6		3	2.8	
>60 (*n* = 44)	28	63.6		17	38.6		11	25.0		10	22.7		4	9.1	
Level of education			0.3			0.3			0.6			0.5			0.3
Low (*n* = 115)	60	52.2		36	31.3		32	27.8		26	22.6		11	9.6	
Middle (*n* = 55)	28	50.9		14	25.5		18	32.7		14	25.5		2	3.6	
High (*n* = 139)	59	42.4		51	36.7		42	33.8		40	28.8		8	5.8	
Profession/occupations			0.5			0.6			0.04			0.4			0.6
Unemployed/student/ other (*n* = 95)	45	47.4		27	28.4		40	42.1		21	22.1		6	6.3	
Worker/salaried/ artisan/merchant/ official (*n* = 134)	65	48.5		44	32.8		33	24.6		33	24.6		12	9.0	
Executive/teacher/liberal profession (*n* = 42)	16	38.1		17	40.5		12	28.6		15	35.7		1	2.4	
Pensioner/retired (*n* = 38)	21	55.3		13	34.2		12	31.6		11	28.9		2	5.3	
Level of anxiety			0.2			0.7			0.07			0.7			0.002
None (*n* = 142)	75	52.8		46	32.4		42	29.6		40	28.2		7	4.9	
Moderate (*n* = 89)	37	41.6		32	36		26	40.4		21	23.6		2	2.2	
High/severe (*n* = 78)	35	44.9		23	29.5		19	24.4		19	24.4		12	15.4	

Note: low level: primary education (8 years or less), medium level: some secondary education (9–11 years), high level: completed secondary education (12 years or more).

**Table 3:** Attitudes and responses of healthcare professionals to gingival bleeding reported by the patients.

Reactions/Responses of Healthcare Professionals	Physician		Dentist		Pharmacist	
	*n*	%	*n*	%	*n*	%
This bleeding is not serious	34	47.9	59	27.3	13	32.5
It is a common and normal phenomenon	4	5.6	6	2.8	1	2.5
Prescription of appropriate mouthwash or toothpaste	20	28.2	119	55.1	18	45.0
Change for a soft-bristle toothbrush	15	21.1	79	36.6	12	30.0
Tooth brushing education	11	15.5	83	38.4	0	0.0
You should see a dentist	19	26.8	NA	NA	10	25.0
Gingival examination	NA	NA	88	40.7	NA	NA

Note: NA = not applicable.
